# Deep Sequencing Reveals Predominant Expression of miR-21 Amongst the Small Non-Coding RNAs in Retinal Microvascular Endothelial Cells

**DOI:** 10.1002/jcb.24084

**Published:** 2012-02-01

**Authors:** Jasenka Guduric-Fuchs, Anna O'Connor, Angela Cullen, Laura Harwood, Reinhold J Medina, Christina L O'Neill, Alan W Stitt, Tim M Curtis, David A Simpson

**Affiliations:** Centre for Vision and Vascular Science, Queen's University BelfastBelfast, Northern Ireland, UK

**Keywords:** microRNA, ENDOTHELIUM, ANGIOGENESIS, DEEP SEQUENCING, RETINA, MICROVASCULATURE

## Abstract

The retinal vascular endothelium is essential for angiogenesis and is involved in maintaining barrier selectivity and vascular tone. The aim of this study was to identify and quantify microRNAs and other small regulatory non-coding RNAs (ncRNAs) which may regulate these crucial functions. Primary bovine retinal microvascular endothelial cells (RMECs) provide a well-characterized in vitro system for studying angiogenesis. RNA extracted from RMECs was used to prepare a small RNA library for deep sequencing (Illumina Genome Analyzer). A total of 6.8 million reads were mapped to 250 known microRNAs in miRBase (release 16). In many cases, the most frequent isomiR differed from the sequence reported in miRBase. In addition, five novel microRNAs, 13 novel bovine orthologs of known human microRNAs and multiple new members of the miR-2284/2285 family were detected. Several ∼30 nucleotide sno-miRNAs were identified, with the most highly expressed being derived from snoRNA U78. Highly expressed microRNAs previously associated with endothelial cells included miR-126 and miR-378, but the most highly expressed was miR-21, comprising more than one-third of all mapped reads. Inhibition of miR-21 with an LNA inhibitor significantly reduced proliferation, migration, and tube-forming capacity of RMECs. The independence from prior sequence knowledge provided by deep sequencing facilitates analysis of novel microRNAs and other small RNAs. This approach also enables quantitative evaluation of microRNA expression, which has highlighted the predominance of a small number of microRNAs in RMECs. Knockdown of miR-21 suggests a role for this microRNA in regulation of angiogenesis in the retinal microvasculature. J. Cell. Biochem. 113: 2098–2111, 2012. © 2012 Wiley Periodicals, Inc.

A healthy retinal microvascular endothelium is essential for maintaining the inner blood–retinal barrier and if perturbed, as occurs, for example, in diabetes, there is an increase in vascular permeability. When endothelial cells are activated by angiogenic signals they migrate and proliferate to form new vessels. If this angiogenic process occurs inappropriately, it can result in serious pathologies such as neovascular age-related macular degeneration (AMD), proliferative diabetic retinopathy, and retinopathy of prematurity. To develop the optimum strategies for manipulating the behavior of endothelial cells in these conditions it is important that we understand the regulatory processes that control endothelial gene expression. Whilst many studies have investigated the role of growth factors and transcription factors, the importance of a new level of regulation mediated by small non-coding RNAs (ncRNA) has only recently been recognized. The chief regulatory small ncRNAs characterized in somatic cells are microRNAs. These are 18–23 ribonucleotides in length and are generated by cleavage of hairpin-containing precursors by the RNase III enzyme Dicer [Kim et al., 2009]. After loading into an RNA-induced silencing complex (RISC) microRNAs direct decay or translational inhibition of multiple target mRNAs which contain partially complementary target sites [Bartel, 2009]. MicroRNAs co-operate with transcription factors to form complex regulatory networks [Martinez and Walhout, 2009] targeting signaling pathways and influencing behavior such as angiogenesis [Anand and Cheresh, 2011].

The essential role of microRNAs in endothelial cells is illustrated by the severe phenotypes observed following their perturbation. Embryonic angiogenesis is impaired in mice lacking active Dicer, presumably due to the loss of microRNA processing [Yang et al., 2005]. Knockdown of Dicer impairs angiogenesis in vitro [Kuehbacher et al., 2007] and deletion of the vascular-specific microRNA miR-126 results in partial embryonic lethality, loss of vascular integrity and delays vascularization of the retina [Fish et al., 2008; Kuhnert et al., 2008; Wang et al., 2008]. The many studies demonstrating a role for microRNAs in either promoting (e.g., miR-126, miR-17-92 cluster) or inhibiting (e.g., miR-221, miR-222) angiogenesis are the subject of several recent reviews [Wang and Olson, 2009; Bonauer et al., 2010; Small and Olson, 2011].

It is the microvasculature which is primarily involved in pathological retinal neovascularization. Primary microvascular endothelial cells isolated from bovine retina (RMECs) maintain many of their in vivo characteristics in culture and have been well characterized by the authors of this study and others and widely used as a model system amenable to experimental manipulation [Stitt et al., 1995]. Thorough knowledge of the small RNAs expressed in RMECs would underpin studies of their function in microvascular endothelial cells. We therefore assessed which approach would be capable of providing the most comprehensive expression profile. Whilst microarrays have been used to measure many microRNAs in parallel in human umbilical vein endothelial cells [Poliseno et al., 2006; Vasa-Nicotera et al., 2011] and RT-qPCR to sensitively measure microRNAs in rat retinal endothelial cells [Kovacs et al., 2011], both techniques share several significant limitations. Due to sequence-specific hybridization properties and variations in PCR efficiency, respectively, quantitative comparisons between different microRNAs are difficult, making it hard to assess their relative likely impacts on gene expression. Also both these approaches require prior sequence information and therefore cannot detect the many novel ncRNAs almost certainly remaining to be discovered in specific tissues and in less well-characterized organisms; only 672 bovine microRNAs have been reported to date in comparison with ∼1,426 in humans (miRBase Release 17, http://www.mirbase.org/ [Kozomara and Griffiths-Jones, 2011]). Finally, these hybridization-based techniques have limited ability to discriminate between closely related sequences, especially isomiRs which may vary in length by only one or two ribonucleotides [Lee et al., 2010].

A deep sequencing approach was therefore chosen to thoroughly characterize the global expression pattern of small ncRNAs, in particular microRNAs, in RMECs. This does not require prior sequence knowledge, provides a digital measure of gene expression and has been used previously to measure small regulatory RNAs in bovine kidney [Glazov et al., 2009]. Highly expressed microRNAs could be prioritized for further study.

The current study reports the expression of a range of small ncRNAs, including sno-miRNAs [Ender et al., 2008; Brameier et al., 2011]. However, microRNAs were the most abundant and included both highly expressed previously reported endothelial microRNAs such as miR-126, miR378, miR-26a and the members of the miR-17-92 cluster and lowly expressed novel microRNAs. The most highly expressed was miR-21, which was demonstrated to play a role in regulation of the angiogenic phenotype of microvascular endothelial cells.

## MATERIALS AND METHODS

### Cell Isolation and Culture

Retinal microvascular endothelial cells (RMECs) were isolated from bovine retina using protocols well-established in our laboratory and demonstrated previously to consistently generate highly pure endothelial cell cultures with minimal contamination [Stitt et al., 1995]. Briefly, bovine eyes were transported from a local abattoir (Ballymena Meats) on ice and the retinas removed and washed free of RPE in Dulbecco's minimal essential medium (DMEM; Life Technologies). The retina was cut into segments, homogenized in MEM and then filtered through an 85 µm filter. The trapped microvessels were digested at 37°C for approximately 30 min in PBS containing 250 µg/ml pronase, 200 µg/ml DNAase, and 50 µg/ml collagenase. The filtrate was microscopically examined to determine the end point for maximum retrieval of endothelial cells. Vessel fragments were then trapped in a 53 µm filter which was centrifuged at 1,100 rpm for 10 min. The pellet was resuspended in Dulbecco's modified Eagles medium (DMEM) containing antibiotic (0.1 mg/ml Primocin), 0.38 µg/ml insulin, 1 mg/ml heparin, and 20% porcine serum (Sigma). This mixture was seeded into 25 cm^2^ gelatin-coated Falcon flasks and maintained at 37°C in a mixture of 5% CO_2_ and air. 10% porcine serum was used in the culture medium from passage 1 onwards and cells were analyzed at passage 2. The cobblestone appearance and characteristic immunolabeling confirmed that these cultures comprise a homogeneous population of cells with endothelial characteristics (Supplementary Fig. 1).

HEK293T cells from the American Type Culture Collection (ATCC) were cultured in DMEM supplemented with 10% fetal calf serum (FCS) and 100 µg/ml Primocin™ (InvivoGen) and were maintained in a humidified incubator containing 5% CO_2_ at 37°C. Human microvascular endothelial cells (HMEC-1) [Ades et al., 1992] were cultured under standard conditions (5% CO_2_, 37°C, 95% humidity) in MCDB 131 medium (Gibco) containing 10% FCS, l-glutamine (5 mM), epidermal growth factor (10 ng/ml), hydrocortisone (1 µg/ml), and antibiotics. As described previously [Medina et al., 2010], OECs were obtained from the mononuclear cell fraction of umbilical cord blood, obtained under full ethical approval from The Office for Research Ethics Committees Northern Ireland (ORECNI). Cells were resuspended in EGM-2 medium (Lonza) supplemented with 10% FCS and seeded on 24-well culture plates pre-coated with rat tail collagen type 1 (BD Biosciences) at a density of 1 × 10^7^ cells/ml.

### Immunocytochemistry

RMECs were fixed with ice-cold methanol for 20 min at −20°C. After blocking with 5% goat serum and 0.1% Tween20 for 1 h at room temperature, cells were incubated overnight at 4°C with the following primary antibodies: Pan-cadherin (rabbit polyclonal, 1/100, Cell Signaling Technology, Danvers, MA); Von Willebrand factor (vWF, mouse monoclonal, ready to use, Abcam, Cambridge, UK); Vimentin (mouse monoclonal, 1/50, Dako, Ely, UK); Alpha smooth muscle actin (α-SMA, mouse monoclonal, 1/50, Dako) and CD14 (mouse monoclonal, 1/50, Santa Cruz, Santa Cruz, CA). After washing with PBS, cells were incubated with appropriate secondary antibodies (1:500 goat anti-mouse or goat anti-rabbit Alexa Fluor488, Life Technologies, Paisley, UK) for 1 h at room temperature. For isolectin B4 staining, cells were blocked in 5% BSA for 30 min, incubated with biotinylated *Griffonia simplicifolia* Isolectin B4 (Vector Laboratories, Peterborough, UK) 1:100 for 1 h, washed with PBS and incubated with streptavidin-FITC (Vector Laboratories) 1:200 for 1 h. The cells were visualized and images captured with an epifluorescence microscope (Nikon, Tokyo, Japan) using Nis Elements (Nikon) software.

### Deep Sequencing

**Fig. 1:**
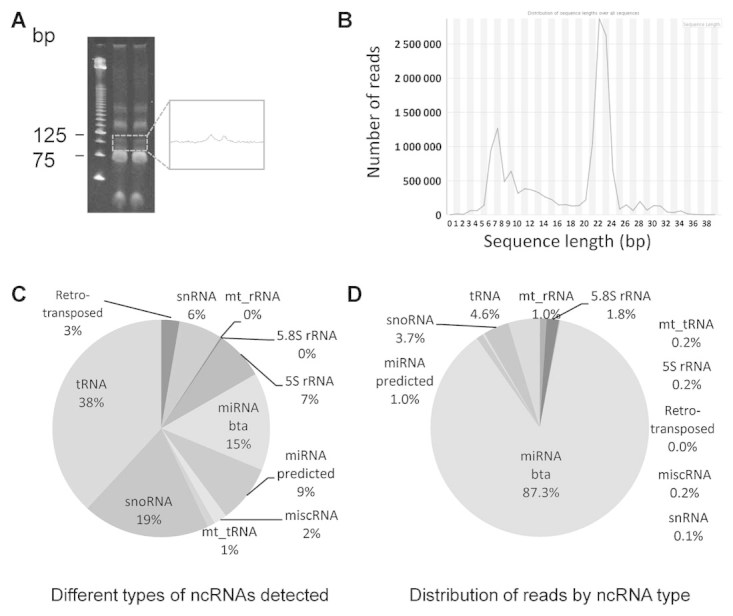
Preparation of small RNA library and general analysis of reads. A: microRNAs (22 nucleotides) with ligated adaptors are 93 nucleotides and the region corresponding to this size was therefore excised from a polyacrylamide gel. The purified small RNA library comprised two distinct bands observed in a Bioanalyzer electropherogram (inset). B: The average length of reads after removal of adapter sequences (22.5 nucleotides) corresponds to that expected for microRNAs. The second main peak at seven nucleotides consists of a mixture of small GC rich inserts. Most of the reads comprising the much smaller peak at 28–30 nucleotides are derived from snoRNAs and tRNA-Gly_GCC. C: Pie chart illustrating the diversity of ncRNAs that contributed reads. The size of each section reflects the number of different members of each class of ncRNAs that was detected. D: Pie chart illustrating the relative abundance of each class of ncRNA as measured by total number of reads aligning to that type of ncRNA. The fact that miRNAs account for >87% of reads but only 15% of the different types of ncRNAs identified means that on average each microRNA is represented by more reads that the other species in our library. miRNA, microRNA; miscRNA, miscellaneous RNAs; mt_rRNA, mitochondrial ribosomal RNA; mt_tRNA, mitochondrial tRNA; snRNA, small nuclear RNA; snoRNA, small nucleolar RNA.

RNA was extracted using a microRNeasy kit (Qiagen). A small RNA library was prepared using a sample prep kit v1.5 kit (Illumina) following the manufacturer's protocol. Briefly, following linker ligation and PCR amplification the range of products corresponding to small RNAs were excised from a 6% polyacrylamide gel (Fig. 1A). Cluster generation and sequencing on a Genome Analyzer II was performed at the Trinity Genome Sequencing Laboratory (http://www.medicine.tcd.ie/sequencing).

### RT-qPCR

Specific microRNAs were detected using a modified version of the method described by Shi and Chiang [2005], in which mature microRNAs are polyadenylated and target sequences for a reverse primer are subsequently incorporated into cDNA by use of an oligo dT adapter. One microgram of RNA was polyadenylated using poly(A) polymerase (PAP; Ambion) at 37°C for 1 h in a 25-µl reaction mixture. RNAs were then reverse transcribed with 200 U reverse transcriptase (SuperScript III; Invitrogen) and 0.5 µg poly (T) adapter (3′ rapid amplification of complementary DNA ends (RACE) adapter in the FirstChoice RLM-RACE kit; Ambion). Primers for specific microRNAs were designed from microRNA sequences obtained from miRBase [Kozomara and Griffiths-Jones, 2011] and from our deep sequencing data (Supplementary Table I). The reverse primer was the 3′ adapter primer (3' RACE outer primer in the FirstChoice RLM-RACE kit; Ambion). RTqPCR was performed with Maxima SYBR Green qPCR mastermix (Fermentas) in 10 µl reactions containing 2 µl of 1:15 cDNA dilution. Reactions were performed on a LightCycler (Roche), with the initial preincubation at 50°C for 2 min and activation at 95°C for 10 min, followed by 40 cycles at 95°C for 15 s, and 60°C for 60 s, with fluorescence acquired after 15 s of the 60°C step. Primer efficiencies were determined using serial dilutions of pooled cDNA and the reactions were optimized to achieve efficiencies ∼2 for all primers used. The RNA from three independent experiments was analyzed and all PCR reactions were performed in triplicates. Gene expression data were normalized to 5S rRNA. The relative expression was determined as 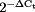, where ΔC_t_ = C_t_(miRNA) − C_t_(5S rRNA). RT-qPCR for a miR-21 target Sprouty1 was performed using the following program; initial denaturation at 95°C for 10 min, followed by 45 cycles at 95°C for 15 s, 58°C for 10 s, and 72°C for 15 s. Data were normalized to GAPDH expression levels. All primer sequences used for qRT-PCR are listed in Supplementary Table I.

### Analysis of Sequence Data

Adapter sequences were clipped from the raw sequences and reads with adapters removed aligned to miRBase (V16) [Kozomara and Griffiths-Jones, 2011] and to the bovine genome (Baylor version Btau_4.0) using Genomics workbench software (CLCbio, Aarhus, Denmark). A parallel analysis was performed using the Galaxy platform http://usegalaxy.org [Blankenberg et al., 2010] and the Bowtie short read aligner [Langmead et al., 2009]. Read lengths were visualized using the Galaxy interface for FastQC. Genomic intervals defined by coverage of aligned reads were displayed graphically as custom tracks in the UCSC genome browser. Ensembl non-coding RNA (ncRNA) annotations for the bovine genome, including predicted microRNAs, were downloaded using Biomart (http://www.biomart.org/). RNA secondary structure was assessed using the Vienna RNAfold webserver (http://rna.tbi.univie.ac.at/). The mirTools web server [Zhu et al., 2010] was used to identify putative novel miRNAs. The co-ordinates of bovine tRNA genes were obtained from the Genomic tRNA database (GtRNAdb: http://gtrnadb.ucsc.edu/Btaur/) [Lowe and Eddy, 1997]. Sequencing data have been deposited in GEO [Barrett et al., 2011] and miRBase [Kozomara and Griffiths-Jones, 2011]. Target genes of miRNAs were predicted using TargetScan (http://www.targetscan.org/) Human release 6.0 or Custom release 5.2 [Lewis et al., 2005]. Gene lists were uploaded to the Database for Annotation, Visualization and Integrated Discovery (DAVID v6.7: http://david.abcc.ncifcrf.gov/) for functional annotation and detection of enriched functional categories [Huang et al., 2009].

### Transfection

RMECs were transfected by electroporation using the Amaxa system (Lonza) with Basic Nucleofector Kit for primary mammalian endothelial cells and M-003 program. Transfection was performed following the manufacturer's protocol for this kit. Briefly, 4 × 10^5^ cells were trypsinized and electroporated with 250 pmol of anti miR-21 miRCURY LNA microRNA inhibitor (Exiqon), miRCURY LNA microRNA negative control, or 3 µg of GFP control plasmid supplied with the kit. Transfected cells were plated on gelatin-coated T25 flasks and left undisturbed for 24 h, when the medium was changed. Cell assays were performed 48 h following electroporation.

### Scratch Wound Migration Assay

Forty-eight hours following transfection 8 × 10^5^ RMECs were plated in a six-well plate previously labeled with lines to facilitate identification of the same regions at different time points. Cells were incubated for 24 h to reach confluency and a scratch was made in the monolayer using a 200 µl pipette tip. Floating cells were removed by two gentle washes with PBS and the growth medium was replaced with DMEM supplemented with 5% porcine serum only to minimize cell proliferation. Reference photographs were taken using a phase contrast microscope Eclipse E400 (Nikon) and the same areas photographed after 16 h. Endothelial cell migration was quantified as the difference in size between the denuded area immediately after the scratch and at 16 h later. NIS elements (Nikon) software was used to measure the area size.

### Tubulogenesis Assay

To facilitate visualization of the tubes, RMECs were labeled with the green PKH67 membrane dye following the manufacturer's protocol (Sigma–Aldrich) and 1 × 10^5^ cells were resuspended in 25 µl DMEM and mixed in a 1:1 ratio with growth factor-reduced Matrigel (BD Biosciences). Fifty-microliter aliquots were spotted onto 24-well plates. After Matrigel polymerization, blobs were covered with DMEM with 5% porcine serum. To analyze tube formation live cultures were imaged 48 and 72 h after plating, using a confocal microscope (Nikon). The tube length was analyzed using NIS elements software (Nikon) and the tube area was measured using Volocity software (Perkin-Elmer).

### Quantification of Cell Proliferation

Transfected RMECs were cultured on gelatin-coated coverslips for 24 h and then fixed in 4% paraformaldehyde for 20 min at room temperature. Blocking was performed for 1 h at room temperature in 10% normal goat serum, 0.3% Triton X-100, in PBS, and incubation with mouse monoclonal Ki67 primary antibody (BD Biosciences), 1:300 dilution, overnight at 4°C. After six washes with PBS, cells were incubated with Alexa Fluor 488 secondary antibody for 1 h at room temperature, and mounted in Vectashield mounting medium with DAPI (Vector labs). Images were collected using an epifluorescent microscope (Nikon). Omission of the primary Ki67 antibody served as a negative control and no staining confirmed absence of non-specific binding.

### Statistical Analysis

Analyses for statistical significance [unpaired *t*-test and one-way analysis of variance (ANOVA) test] were carried out using Prism 4.0 software (GraphPad Software, San Diego, CA). Statistical significance was set as *P* < 0.05.

## RESULTS

Raw data have been deposited in Gene Expression Omnibus (GEO) with accession number GSE31340 and the novel microRNAs have been deposited in miRBase.

Small RNA library preparation was performed using total RNA extracted from RMECs (Fig. 1 A, Supplementary Fig. 1). Following removal of adapter sequences from the 20,449,761 raw reads, the highest peak in the size distribution of the reads was at the length expected for microRNAs (21–24 nucleotides; Fig. 1 A,B). However, many reads were too short to represent microRNAs and only the 10,608,130 reads of >15 nucleotides in length were analyzed further (the peak at 7 nucleotides corresponding to the shorter band on the electropherogram consists of a mixture of small GC rich inserts). These >15 nucleotide reads were collapsed into a list of 320,426 unique sequences. Those reads present multiple times are less likely to be due to sequencing errors and therefore more likely to represent endogenous RNAs. Removal of sequences represented by <3 reads reduced the number of unique sequences significantly to 71,900, whilst still accounting for 97% of all reads [a cut-off of >10 reads resulted in a list of 16,948 unique reads which could be more readily presented as supplemental data (Supplementary Table II)]. The aim of applying this crude filter of 3 reads was to remove many likely artifactual sequences whilst retaining most of the bona fide endogenous RNA-derived sequences. The sequences represented by at least 3 reads mapped to 1,208 different ncRNAs annotated by Ensembl or the Genomic tRNA database (Fig. 1C). However, whilst this broad range of ncRNAs was detected, microRNAs were by far the most highly expressed group, accounting for 87.3% of all mapped reads (Fig. 1D).

**Fig. 2:**
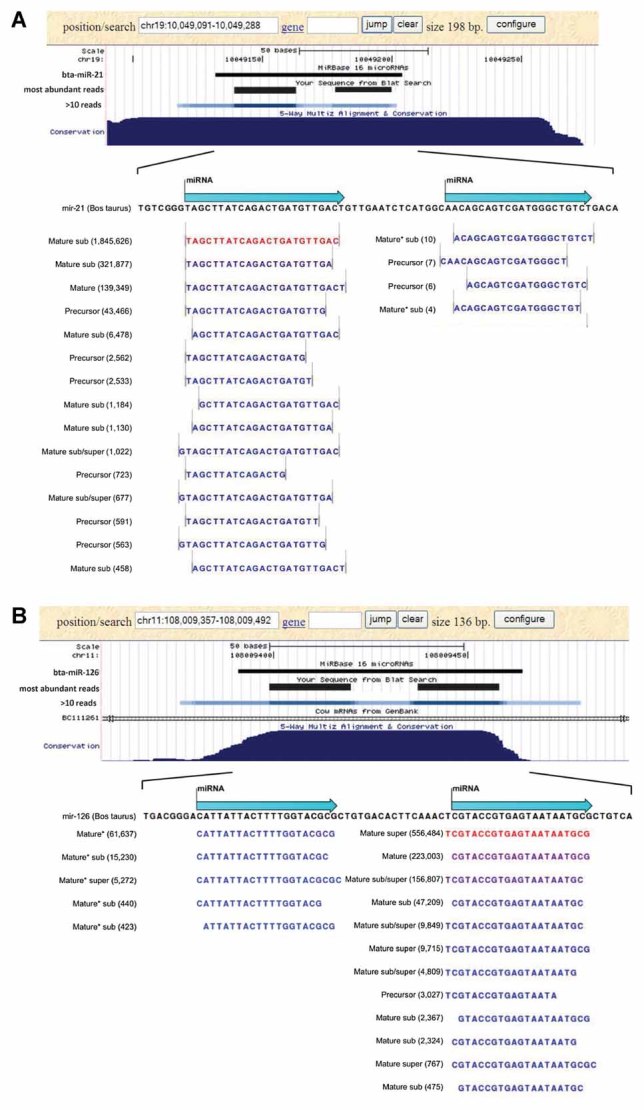
IsomiRs and detection of star sequences. A: The region of chromosome 19 encoding pre-miR-21 (bta-mir-21) is depicted in the UCSC genome browser at the top of the figure. The dark black bands indicate the positions of the two most abundant sequences, while the intensity of shading of the line below is proportional to the number of reads spanning each position (minimum cut-off 10 reads). The multiple alignment with dog human, mouse and platypus shows that this gene is highly conserved. The central region is expanded to show the sequences and numbers of aligned reads which comprise isomiRs of miR-21 and its star sequence (only those reads with >400 and >3 occurrences for miR-21 and the star sequence, respectively, are shown). The most frequent isomir of miR-21 is one nucleotide shorter than that reported in miRBase (The miRBase sequence is labeled “mature”: those shorter “sub” or “precursor”; those longer “super”). B: The reads representing miR-126 (>400 occurrences) are depicted as described above for miR-21. The most abundant isomiR again varies from miRbase but in this case by one nucleotide at the 5′ end. Processing is specific for the mature miR-126 microRNA, but the star sequence is more abundant than for miR-21. [Color figure can be seen in the online version of this article, available at http://wileyonlinelibrary.com/journal/jcb]

Alignment to miRBase (release 16) [Kozomara and Griffiths-Jones, 2011] identified 250 bovine microRNAs represented by at least 3 reads (considering mature sequences potentially derived from several distinct precursor sequences as one). Those microRNAs with >1,000 reads per million mapped are shown in Table I and all hits are listed in Supplementary Table III. The most highly expressed microRNA was miR-21 with one-third of all mapped reads, followed by let-7f and miR-126, each with ∼15% of all reads. The 4 most abundant tags representing miR-21 isomiRs exhibited 3′ heterogeneity, but processing was incredibly specific for the mature microRNA, with only 27 reads representing the other strand or “star” sequence compared with 2,318,344 for miR-21 itself (Fig. 2A). To confirm the existence of the isomiRs RT-PCR was performed with primers either matching the full length of the published miR-21 sequence or being shorter by 1 or 2 nucleotides. The shortest primers would be expected to amplify cDNA from all isomiRs, whilst the longer primers would not be expected to amplify cDNA from the shorter isomiRs. Therefore, if the isomiRs exist, lowest expression levels would be detected with the longest primer and highest expression with the shortest primer. This pattern was indeed observed (Supplementary Fig. 2). The reads representing the endothelial-specific miR-126 [Fish et al., 2008; Kuhnert et al., 2008; Wang et al., 2008] also indicate the presence of isomiRs, but the distribution of reads between miR-126 and its star sequence, processed from the other arm of the pre-microRNA is less extreme (1,014,272 vs. 83,798; Fig. 2B). IsomiRs were observed for most of the microRNAs detected and in many cases the most abundant form differed from that reported in miRBase.

**Table I:** Bovine microRNAs Represented by >1,000 Reads per Million Mapped

Name	Total number of reads	Reads per million mapped
mir-21	2,318,344	338,603.3
let-7f-2//let-7f-1	1,073,705	156,818.9
mir-126	1,014,272	148,138.4
let-7a-1//let-7a-2//let-7a-3	387,336	56,572.0
mir-378//mir-378-2	340,689	49,759.0
mir-24-2	258,546	37,761.7
mir-103-1//mir-103-2	148,228	21,649.3
mir-30a	112,865	16,484.4
mir-27b	104,682	15,289.2
let-7g	66,479	9,709.5
mir-452	61,833	9,031.0
mir-30d	61,224	8,942.0
mir-151	60,052	8,770.8
mir-140	55,008	8,034.1
mir-20a	48,329	7,058.6
mir-23a	45,707	6,675.7
mir-17	45,449	6,638.0
let-7b	34,754	5,076.0
mir-148b	33,677	4,918.7
mir-26a-2//mir-26a-1	30,576	4,465.7
mir-191	27,688	4,043.9
mir-93	27,177	3,969.3
mir-184	25,142	3,672.1
mir-186	25,078	3,662.7
let-7e	21,635	3,159.9
mir-29a	21,407	3,126.6
mir-152	20,604	3,009.3
mir-30e	19,696	2,876.7
mir-101//mir-101-1	19,218	2,806.9
let-7i	18,731	2,735.7
mir-423	16,993	2,481.9
mir-28	15,266	2,229.7
mir-155	14,390	2,101.7
let-7c	14,020	2,047.7
mir-143	12,204	1,782.4
mir-181a-2//mir-181a-1	11,882	1,735.4
mir-146a	10,952	1,599.6
mir-15a	9,938	1,451.5
mir-99b	9,755	1,424.8
mir-148a	9,502	1,387.8
mir-125b-1//mir-125b-2	9,395	1,372.2
mir-106b	9,284	1,356.0
mir-26b	9,146	1,335.8
mir-16b	8,750	1,278.0
let-7d	8,273	1,208.3
mir-16a	8,007	1,169.5
mir-92//mir-92a	7,427	1,084.7
mir-7//mir-7-2//mir-7-1	6,852	1,000.8

**Fig. 3:**
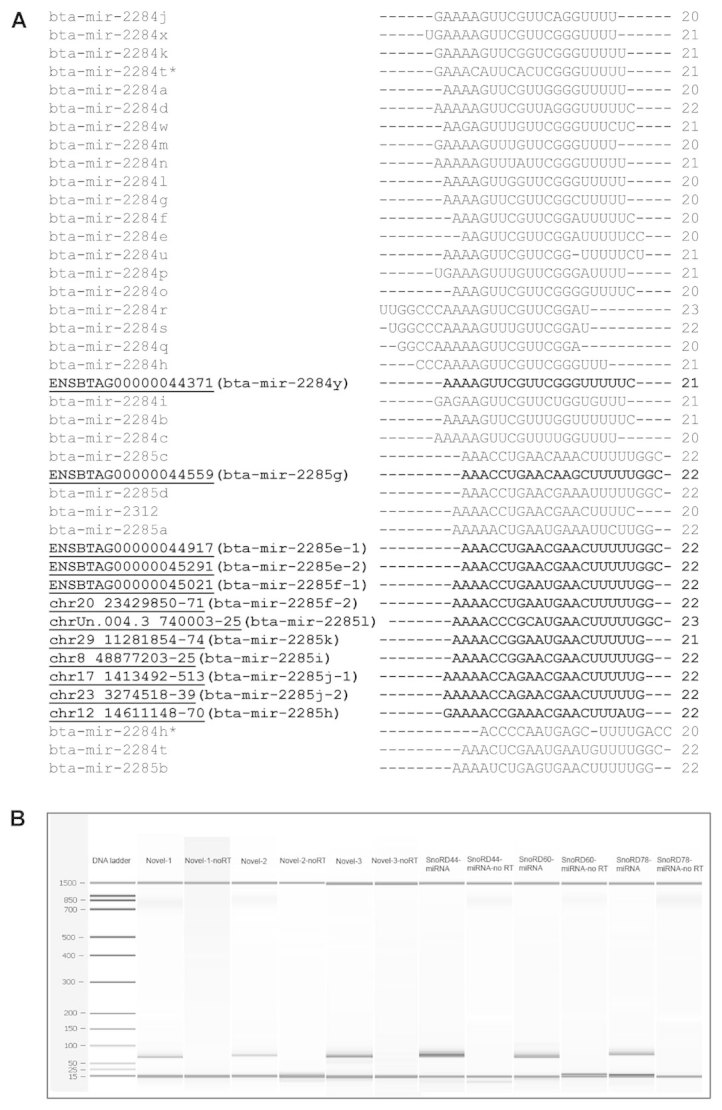
Orthologs and novel microRNAs. A: Multiple alignment of miRNAs in the miR-2284/2285 family. Previously reported members are identified by their miRBase number, whilst novel members detected in this study are highlighted in bold and given their Ensembl predicted gene number or chromosomal location (underlined) followed by their new miRBase number. B: A digital Bioanalyzer gel displaying bands for novel microRNAs and sno-miRNAs amplified by RT-PCR. There was no amplification from templates in which the reverse transcriptase enzyme was omitted (lanes labeled No RT).

Alignment of reads with human microRNAs in miRBase identified 13 previously unreported bovine orthologs of human microRNAs and showed a microRNA previously recorded as the unique family member in cows (bta-miR-450) to be expressed with an other member of this family (Supplementary Table IV). Multiple reads confirmed the existence of four new members of the miR-2284 and miR-2285 families, encoded by five putative Ensembl microRNAs, predicted by alignment of microRNA sequences from miRBase to the bovine genome and evidence of potential stem-loop structures (Fig. 3A and Supplementary Table IV).

Strong evidence for 4 novel microRNAs was uncovered by miRTools [Zhu et al., 2010], which identifies potential precursor stem-loop structures supported by both mature and star sequences (details of these novel microRNAs, are listed in Supplementary Table V). The identification of additional novel microRNA genes for which star sequences are not present is more difficult. To select the best candidates, regions which did not overlap any annotated ncRNA but that had strong evidence of expression (>100 reads) and the optimum size expected for microRNAs (21–23 nucleotides) were analyzed for potential secondary structure. Filtering for those with a predicted free energy of <25 kcal/mol and relatively tight binding of the mature miRNA to one side of a single hairpin, that is, adhering to Ambros' criteria [Ambros et al., 2003], identified eight more novel candidate microRNAs, of which seven are members of the bta-miR-2284/2285 family (Fig. 3; Supplementary Table IV). The predicted target genes of the novel microRNAs and the enriched functional categories are listed in Supplementary Tables VI and VII. The targets of all the novel 2284/2285 family members were predicted from their seven unique seed sequences and a combined list of potential target genes prepared. This was enriched for genes with a range of regulatory functions (Supplementary Table VI).

**Fig. 4:**
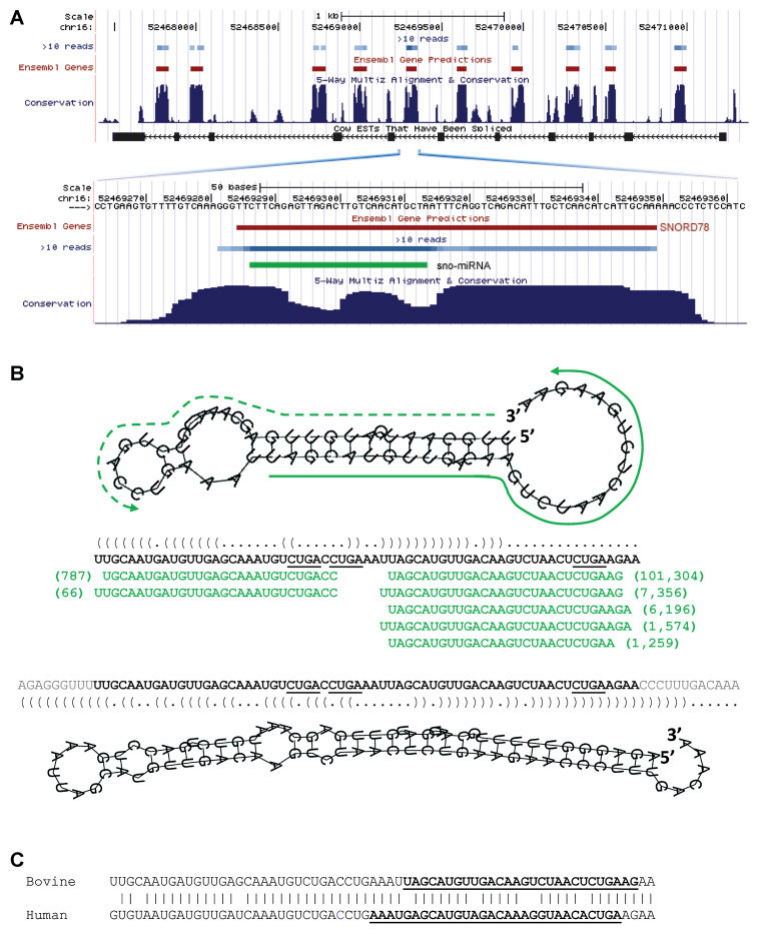
sno-microRNAs. A: A cluster of highly conserved snoRNAs (labeled Ensembl genes) on chromosome 16 (52,468,591-52,471,183) is depicted in the UCSC genome browser. The snoRNAs are transcribed as introns of a non-coding transcript detected as spliced EST sequences. Small RNA sequences derived from all these snoRNAs were observed in our deep sequencing data from primary endothelial cells. The locations of small RNAs represented by >10 reads are indicated with the intensity of shading being proportional to the number of reads spanning each position. The bovine orthologue of the U78 snoRNA (SNORD78) as annotated in Ensembl is expanded to show the position of the predominant novel sno-miRNA represented by 101,948 reads. B: Predicted SNORD78 secondary structure indicating the position of the major sno-miRNAs relative to the predicted stem loop. The secondary structure of the snoRNA is also shown in dot-bracket notation (CUGA D and D' boxes are underlined) with the individual reads depicted beneath (>10 for minor product and >1,000 for main sno-miRNA due to space restrictions). The more stable secondary structure loop, with minimum free energy of −20.5 kcal/mol, predicted to form from an RNA spanning the conserved region and slightly longer than the predicted snoRNA is also shown. C: Bovine and human SNORD78 sequences with the sno-miRNAs underlined. [Color figure can be seen in the online version of this article, available at http://wileyonlinelibrary.com/journal/jcb]

Several studies have reported the existence of snoRNA-derived molecules with microRNA-like function, termed sno-miRNAs [Ender et al., 2008; Brameier et al., 2011] and the snoRNA MBII-52 (SNORD 115) has been shown to be processed into smaller RNAs (termed psnoRNAs) that regulate alternative splicing [Kishore et al., 2010]. We therefore looked for reads indicating the existence of small RNAs originating from snoRNAs. This revealed 369 snoRNAs represented by at least 3 reads (Supplementary Table VIII). The most abundant small RNAs were derived from U78 (SNORD78) with >100,000 identical reads from one isoform accounting for most of the 28 bp reads observed (Fig. 1 B). The equivalent sno-miRNA has been reported previously in humans (Fig. 4C) and shown to be efficient in gene silencing (Brameier et al., 2011). In common with conventional microRNAs, sno-miRNAs can be processed from both arms of the precursor hairpin, and this feature was observed for SNORD78 (Fig. 4B) and most of the other snoRNAs. The SNORD78 gene is located in a cluster of highly conserved snoRNAs which are processed from the introns of the growth arrest-specific transcript 5 (*GAS5*) ncRNA in humans and mice [Smith and Steitz, 1998] and the orthologous transcript, represented by bovine ESTs (e.g., GenBank accession: EE23805) is shown in Figure 4A. Whilst the sno-miRNA from SNORD78 was the most abundant, reads mapped to all the snoRNAs in this cluster, suggesting that these are also processed to form sno-miRNAs.

The presence of three novel microRNAs and of sno-miRNAs derived from SNORD78, SNORD60, and SNORD44 was confirmed by RT-PCR (Fig. 3B).

We observed small RNAs derived from tRNAs, similar to those reported previously [Cole et al., 2009]. The three most abundant reads (36,604, 36,144, and 21,632 occurrences) all match the 5′ end of a tRNA-GlyGCC isoform potentially transcribed from nine genes (the shortest also matching the predicted transcripts from an additional 11 tRNA-GlyGCC genes). The significance of this observation is not clear, but it should be noted that in cows tRNA-Gly genes comprise 44% of all the candidate tRNA genes predicted by tRNAscan [Lowe and Eddy, 1997]. The sequence of miRNAs can be altered by RNA editing and we observed a previously described example of A-to-G transitions potentially caused by A-to-I editing in miR-99a (3% of 752 reads) [Kawahara et al., 2007; Jin et al., 2009].

**Fig. 5:**
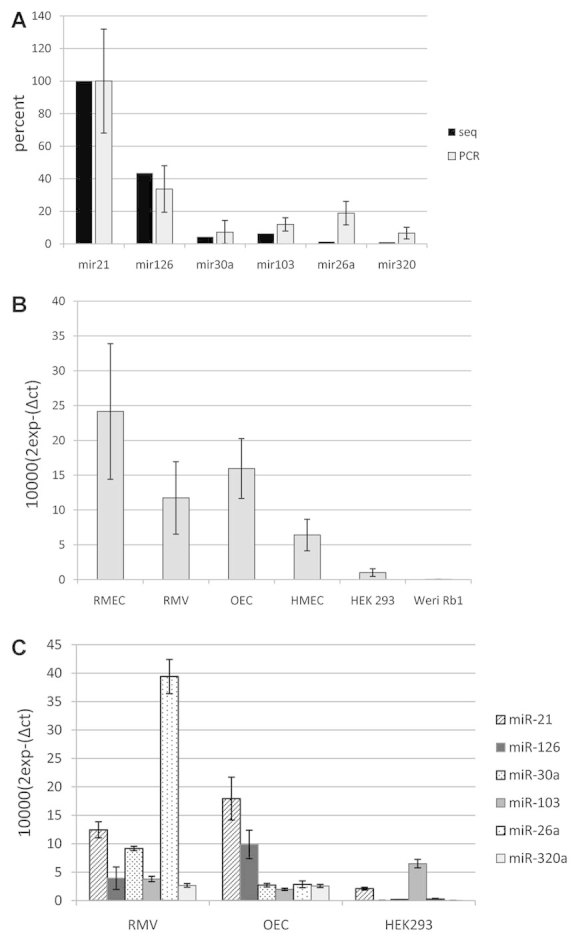
Relative expression of microRNAs measured from sequencing reads or RT-qPCR. A: Comparison of expression of selected microRNAs measured by sequencing (reads per million mapped) or RT-qPCR (relative expression) in RMECs. B: Relative quantification of miR-21 in the cultured primary RMECs, freshly isolated retinal microvessels (RMV), outgrowth endothelial cells (OECs), human microvascular endothelial cells (HMEC-1 cell line), HEK293T and retinoblastoma (Weri-Rb1) cell lines. The data are the mean of three biological replicates (± SD). C: Expression of microRNAs shown in (A) in RMVs, OECs, and HEK293T cells.

The expression of six microRNAs represented by various numbers of reads was assessed by RT-qPCR in biological replicates and compared with the sequencing data (Fig. 5A). PCR results supported the sequencing data and confirmed the extremely high expression of miR-21 relative to other microRNAs. It has been reported that miR-21 is highly expressed in various cancers and its expression is often associated with cell proliferation [Krichevsky and Gabriely, 2009; Selcuklu et al., 2009]. Although expression of miR-21 has been reported previously in endothelial cells, we wanted to exclude the possibility that the extremely high level of miR-21 in RMEC is due to cell proliferation in vitro. We therefore performed RT-qPCR on freshly isolated bovine retinal microvessels prior to culture and also examined the expression of miR-21 in other endothelial and cancer cell lines. The expression of miR-21 was also high in the native microvascular tissue, at approximately half the levels observed in primary cultures of RMECs (Fig. 5B). miR-21 was also highly expressed in the two other endothelial cell types assessed, human microvascular endothelial cells (HMEC-1 cell line [Ades et al., 1992]) and outgrowth endothelial cells (OECS [Medina et al., 2010]). In contrast, miR-21 expression was significantly lower in rapidly dividing, non-endothelial cell lines; immortalized kidney (HEK293T) and retinoblastoma (Weri-Rb1; Fig. 5B). The relative expression levels of the other five microRNAs in RMVs and OECs were also similar to those in RMECs, with the exception of miR-26a (Fig. 5C).

**Fig. 6:**
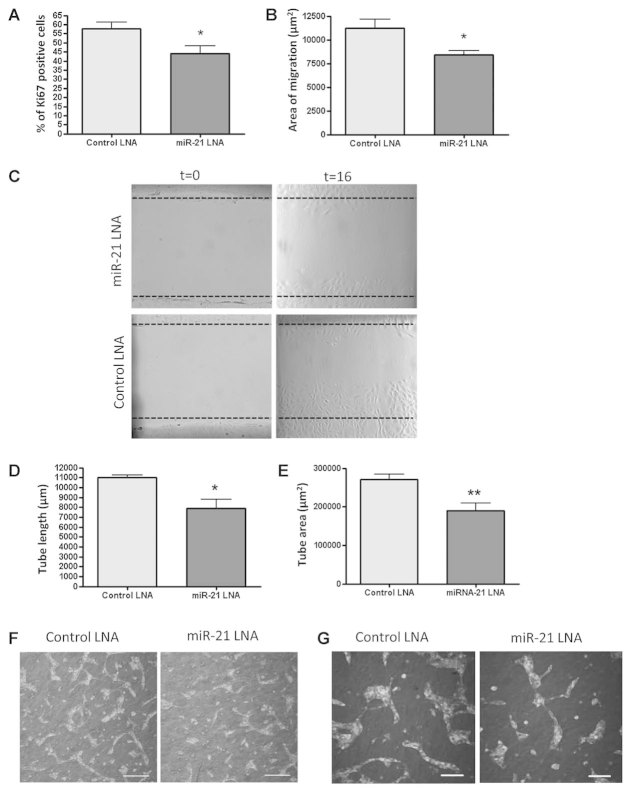
Inhibition of miR-21 reduces angiogenic properties of RMECs. RMECs were assessed for proliferation, migration and tubulogenesis 48 h post-transfection with miR-21 LNA inhibitor or LNA scrambled control. A: Cell proliferation determined by immunocytochemistry for proliferation marker Ki67. Cells were plated on gelatin-coated coverslips and cultured for 24 h in DMEM supplemented with insulin, heparin, and 10% porcine serum. Ki67 positive and negative cells were counted in 6 random fields, n = 4 per group. B: Quantification of cell migration of transfected RMECs in a scratch-wound assay. A scratch was made in confluent cultures and cells were maintained in DMEM supplemented with 5% porcine serum for 16 h. The size of the denuded area immediately after the scratch (t = 0) was compared to the size of the area 16 h later to estimate cell migration. Four fields were analyzed; n = 4 per group. Representative images are shown in (C). D: Quantification of the tube length of transfected RMECs cultured on Matrigel for 48h. E) Quantification of the tube area after 72 h of Matrigel culture. Three fields were analyzed, n = 4 per group. Cells were labeled with PKH67 before plating on Matrigel and maintained in DMEM supplemented with 5% porcine serum. Representative images of tube formation after 48 h are shown in (F) and after 72 h in (G). Data are expressed as mean ± SEM, *P < 0.05, **P < 0.01 versus corresponding control determined by unpaired *t*-test. Scale bar in (F) is 200 µm and in (G) 100 µm.

Analysis of the functions of predicted miR-21 target genes revealed enrichment of regulatory genes, with one annotation cluster (no. 10) relating specifically to angiogenesis (Supplementary Table IX). To gain experimental insights into the function of miR-21 in RMECs, its activity was attenuated by transfection with a miR-21 LNA inhibitor (Exiqon; Supplementary Fig. 3). Cells transfected with a scrambled LNA sequence served as control. The effects of miR-21 inhibition on the three basic components of angiogenesis: cell proliferation, migration and tube formation were assessed. To estimate cell proliferation, RMECs were cultured on gelatin-coated coverslips for 24 h and fixed for immunocytochemistry. Inhibition of miR-21 reduced cell proliferation, as determined by percentage of cells immunolabeled for the proliferation marker Ki67 (Fig. 6A). RMECs transfected with anti-miR-21 also displayed decreased cell migration in the scratch wound assay (Fig. 6B,C). A scratch wound was made on confluent monolayers of transfected RMECs and the denuded area imaged and measured at time 0. The size of the denuded area was again measured 16 h later and the migration was estimated as difference between denuded area at t = 16 and t = 0. Finally, we investigated the ability of RMECs to form a tube network when plated on Matrigel extracellular matrix. Both, the total tube length (Fig. 6D,F) and the tube area (Fig. 6E,G) were reduced in anti-miR-21 transfected cells after 48 and 72 h, respectively, of culture on Matrigel.

## DISCUSSION

The ability of many small RNAs to regulate cellular physiology has been widely demonstrated, particularly in the cardiovascular system [Small and Olson, 2011]. The detection of a wide range of non-coding RNAs in RMECs in this study underlines the advantage of sequencing-based expression profiling. Small RNAs derived from snoRNAs and tRNAs have been reported in other tissues [Ender et al., 2008; Cole et al., 2009; Taft et al., 2009; Brameier et al., 2011]. The sno-miRNAs we detected were approximately 30 nucleotides in length, similar to piwi-interacting RNAs in germ cells. Whilst the functional significance of these non-microRNA small RNAs is unclear, the potential activity of sno-miRNAs has been demonstrated (Brameier et al., 2011). Almost all the snoRNAs which were identified by Brameier et al. as sources of sno-miRNAs in CD4+ T lymphocytes also had abundant processed small RNAs in RMECs. The high expression of sno-miRNAs, particularly sno-miRNA78, makes it a priority to establish their function in RMECs.

As was the case in a study of bovine kidney [Glazov et al., 2009], our sequence data have enabled identification of novel small RNAs and the discovery of new bovine members of known microRNA families. These findings contribute to the rapidly increasing catalog of small RNAs available in miRBase [Kozomara and Griffiths-Jones, 2011] and confirm other studies which suggest that many novel microRNAs remain to be discovered. The high depth of coverage enabled the detection of isomiRs for almost all the microRNAs detected, supporting previous reports of small RNA complexity [Lee et al., 2010]. Although it cannot be ruled out that some of these sequences are cloning artifacts, it is widely accepted that they largely reflect endogenous variants [Lee et al., 2010]. The predominant isomiR of miR-21 was one base shorter than the mature sequence listed in miRBase. The demonstration that RT-PCR employing a primer based upon the published mature sequence detected less microRNAs than shorter primers which amplified more isoforms illustrates a potential source of error with this approach to quantification. Whilst minor variations in length at the 3′ end may have little functional significance, the rarer 5′ variants are more likely to have altered target genes.

The microRNAs detected were consistent with previous studies of endothelial cells. Approximately 40% of the 100 most highly expressed microRNAs are the same in rat retinal endothelial cells [Kovacs et al., 2011] and bovine RMECs. The well-characterized endothelial-specific miR-126 was the third most highly expressed microRNA. Other highly expressed microRNAs previously associated with endothelial cells included miR-21, miR-378, miR-20a, miR-17, and miR-26a [Kuehbacher et al., 2007; Wang and Olson, 2009; Bonauer et al., 2010]. Many of the unique features of the microRNA profile are likely to be due to the microvascular origin of the RMECs. Macro- and micro-vascular endothelial cells have distinct functional properties and mRNA expression patterns [Bhasin et al., 2010] and these appear to be reflected in microRNA expression. Several of the top microRNAs in RMECs, for example, miR-452 and miR-151, have not previously been reported as highly expressed in endothelial cells.

In addition to biological variation between species or endothelial cell types, the differences in microRNA expression observed relative to previous studies could in part be attributable to the methodology. Although not susceptible to variations in hybridization efficiency, the levels of expression detected by sequencing are potentially prone to differences in adaptor ligation efficiency during library preparation, which could skew the number of reads detected for different sequences [Hafner et al., 2011]. It is therefore important to assess microRNAs by more than one method and the broad agreement observed between sequencing and RT-qPCR increases confidence in the measured levels.

The most highly expressed microRNA in RMECs was miR-21, accounting for 34% of all mapped reads. Although functional assessment of miR-21 in various cell types has revealed regulatory roles in processes such as cell proliferation, migration, and apoptosis, perhaps surprisingly genetic deletion of miR-21 in mice did not result in any obvious phenotype [Patrick et al., 2010]. Whilst shown previously to be highly expressed in endothelial cells, miR-21 has also been widely associated with cancer state and increased cell proliferation and is the subject of several reviews [Krichevsky and Gabriely, 2009; Selcuklu et al., 2009]. The pro-angiogenic role for miR-21 in RMECs suggested by the decreased proliferation, migration and tube formation observed after its inhibition is in agreement with its reported effects in, for example, glioblastoma [Gaur et al., 2011] and bladder cancer cells [Tao et al., 2011]. Indeed miR-21 has been shown to induce prostate tumor angiogenesis by targeting PTEN and increasing HIF-1 and VEGF expression [Liu et al., 2011]. Intriguingly, in human umbilical vein endothelial cells (HUVECs) miR-21 was reported to have antiangiogenic properties by targeting RhoB [Sabatel et al., 2011]. The dependence of miR-21 function upon specific cellular environment presumably reflects different target mRNA expression, and is consistent with previous studies showing that micro- and macro-vascular endothelial cells also respond differently to insulin [King et al., 1983] and glucose [Duffy et al., 2006]. High-glucose treatment decreased cell viability and induced apoptosis in human aortic endothelial cells (HAECs), but had an opposite effect on human retinal endothelial cells, leading to increased viability and inhibition of apoptosis [Duffy et al., 2006]. The high expression of miR-21 detected in freshly isolated retinal microvessels suggests an important role for miR-21 in the native vascular endothelium. The high level of miR-26a in RMVs, which is not maintained in RMECs, may be related to its function in cell cycle arrest [Kota et al., 2009].

This study has demonstrated the utility of deep sequencing for profiling small RNA expression, particularly in less well-characterized organisms. The ability to identify candidates for further investigation is exemplified by the discovery of the pro-angiogenic role of miR-21 in the retinal microvasculature.

## Supplementary material

Supplementary Figure 1: Confirmation of endothelial characteristics of primary RMEC isolations. RMECs display the cobblestone morphology typical of endothelial cells when viewed under phase contrast (A). The pattern of immunolabelling observed with a range of antibodies confirmed that RMECs are a very homogeneous population of cells with endothelial characteristics: Both controls with secondary antibody were negative (B and C). All RMECs bound Isolectin (D), formed adherens junctions through cadherin (note negative staining in free edge of cells, E), and expressed vWF (F). Being an intermediate filament protein characteristic of mesenchymal cells, Vimentin serves as a positive control and a strong signal was observed (D). α-SMA was absent in the cultures, which excludes smooth muscle contamination and is consistent with the absences of pericytes. CD14 was negative, suggesting that there is no contamination with haematopoietic cells or macrophages. Scale bar: 100 μm.

Supplementary Figure 2: miR-21 RT-PCR. RT-qPCR performed using primers for miR-21 of varying lengths at the 3' end (22, 23 or 24 nucleotides). The level of expression detected decreased incrementally with primer length, which is consistent with an inability of the longer primers to amplify the shorter isomiRs.

Supplementary Figure 3: RT-qPCR analysis of miR-21 knockdown. A) RT-qPCR confirmed that expression of miR-21 is reduced following transfection with LNA miR-21 miRCURY LNA microRNA inhibitor (Exiqon) relative to expression following transfection with the miRCURY LNA microRNA negative control (p<0.01 unpaired t test). B) Conversely, as expected if miR-21 activity is reduced, the mRNA expression of the miR-21 target gene Sprouty1 is increased following knockdown of miR-21 with LNA inhibitor (p<0.01 unpaired t test).

Supplementary Table 1: Primer sequences

Supplementary Table 2: Sequences represented by ≥ 10 reads

Supplementary Table 3: Known microRNAs represented by ≥ 3 reads

Supplementary Table 4: Novel bovine orthologs of known microRNAs

Supplementary Table 5: Novel microRNAs

Supplementary Table 6: Predicted target genes of the novel miR-2284/5 family members and functional annotation

Supplementary Table 7: Predicted target genes of novel microRNAs, with functional annotation

Supplementary Table 8: Sno-miRNAs

Supplementary Table 9: Predicted target genes of miR-21 with functional annotation
