# Supplementary figures and images for: Deep Sequencing Reveals Predominant Expression of miR-21 Amongst the Small Non-Coding RNAs in Retinal Microvascular Endothelial Cells
Source: J Cell Biochem. 2012 Feb 1;113(6):2098–111. doi: 10.1002/jcb.24084 (PMC3708110; doi:10.1002/jcb.24084)

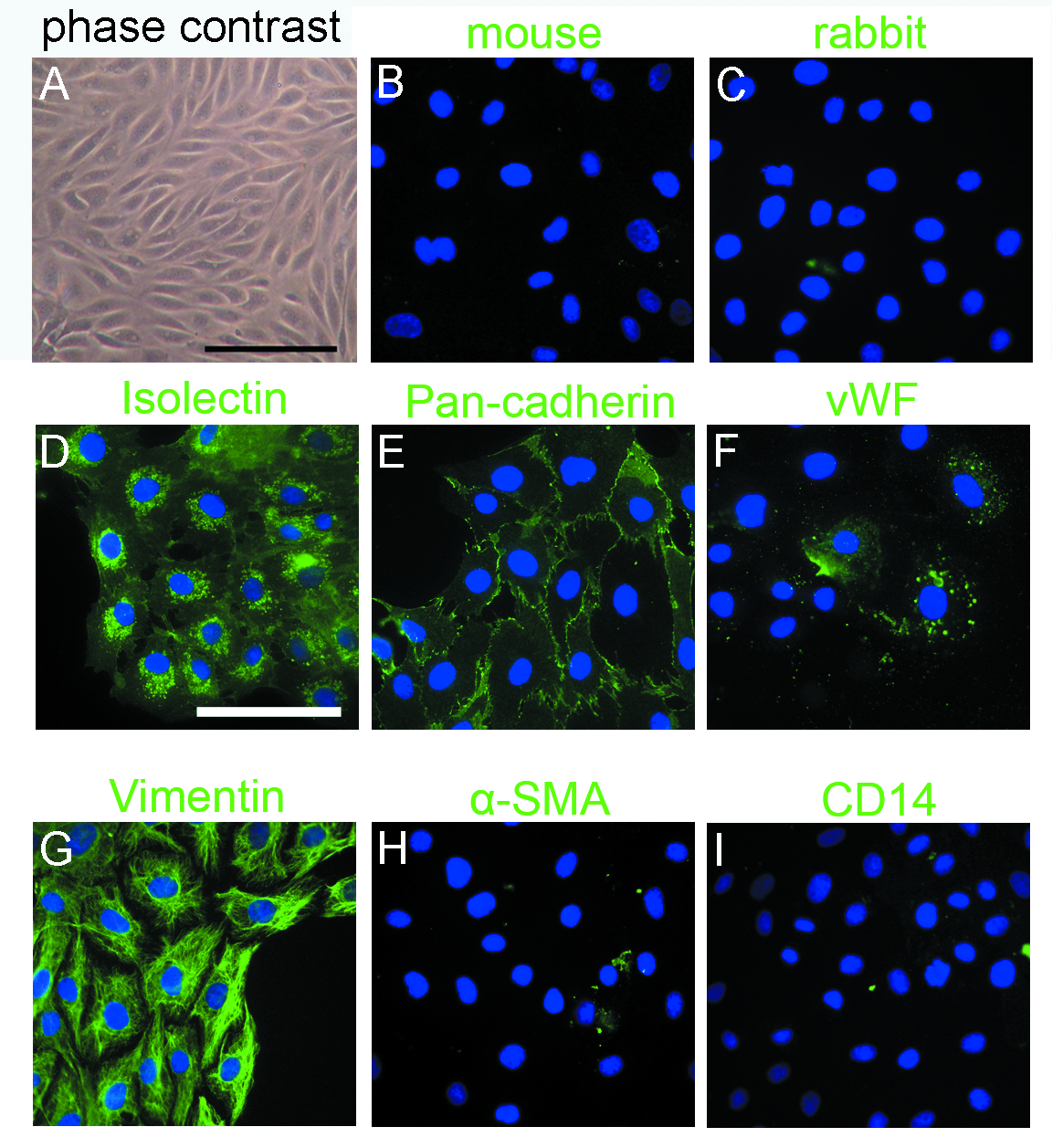

Supplement: Supplementary file 10 [file jcb0113-2098-SD10.tif]

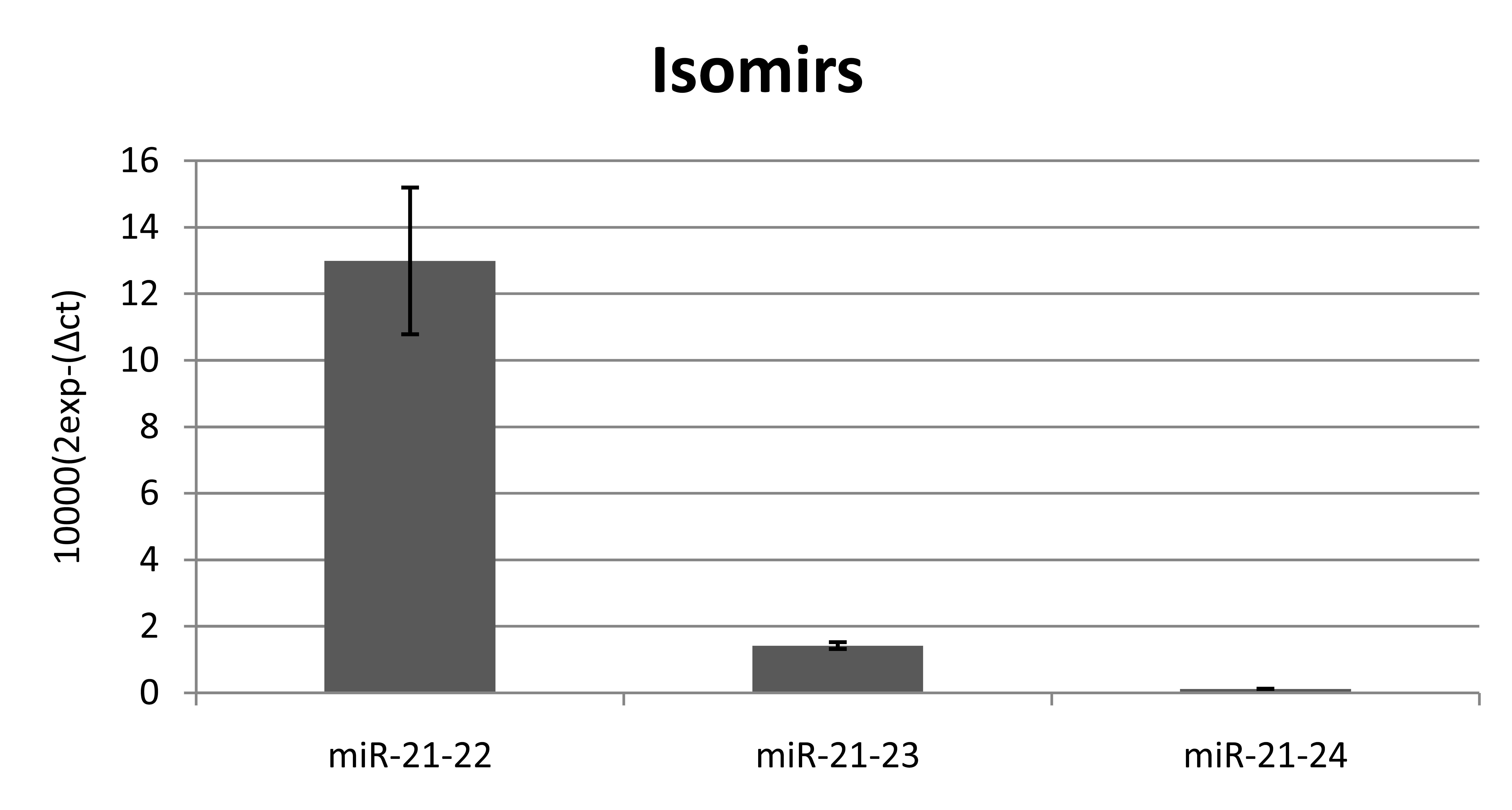

Supplement: Supplementary file 11 [file jcb0113-2098-SD11.tif]

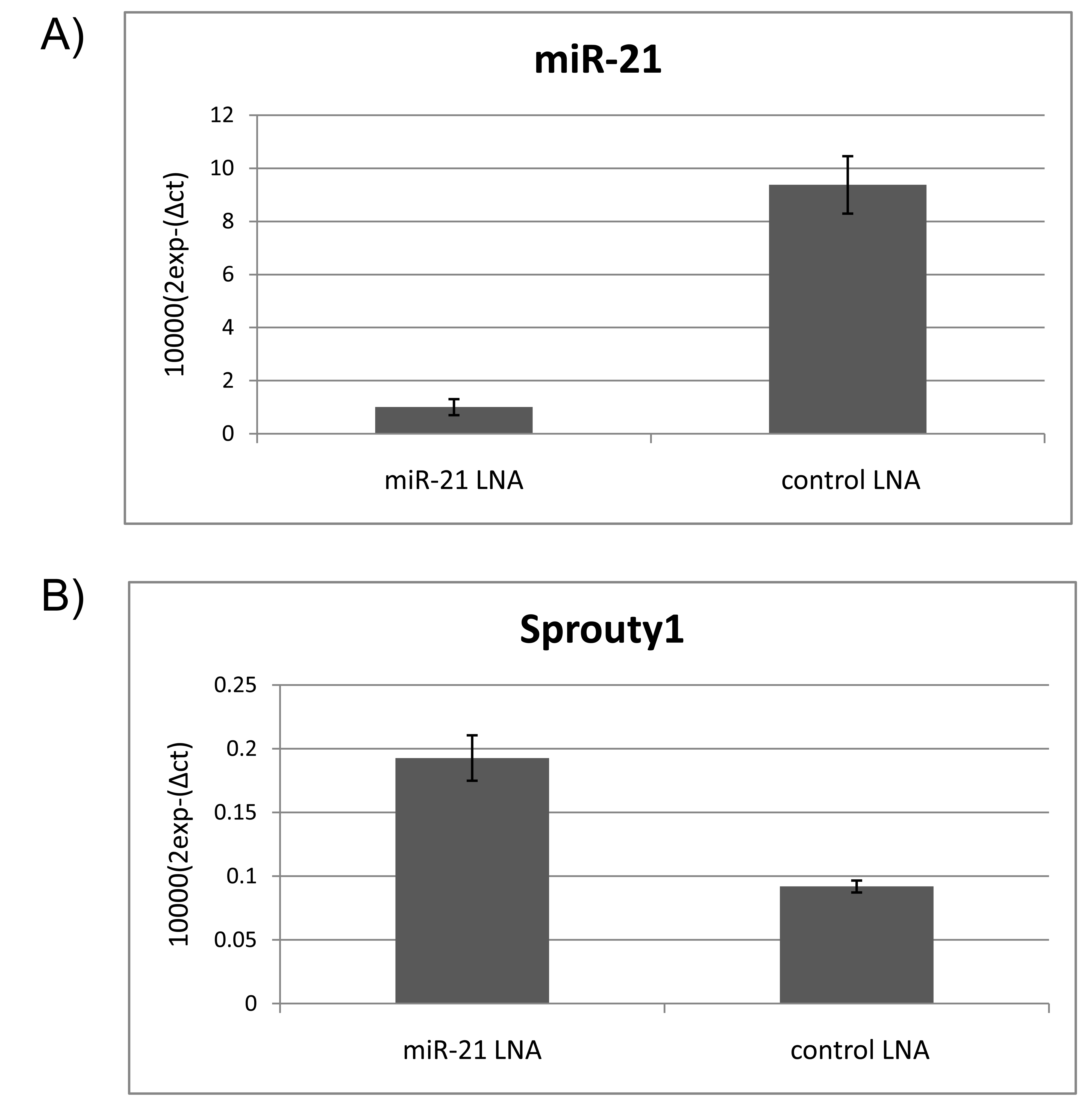

Supplement: Supplementary file 12 [file jcb0113-2098-SD12.tif]
